# Clinical Significance of Serum Albumin/Globulin Ratio in Patients With Pyogenic Liver Abscess

**DOI:** 10.3389/fsurg.2021.677799

**Published:** 2021-11-30

**Authors:** Jia Zhang, Tao Wang, Yi Fang, Mengzhou Wang, Wuming Liu, Junzhou Zhao, Bo Wang, Zheng Wu, Yi Lv, Rongqian Wu

**Affiliations:** ^1^National Local Joint Engineering Research Center for Precision Surgery & Regenerative Medicine, First Affiliated Hospital of Xi'an Jiaotong University, Xi'an, China; ^2^Department of Hepatobiliary Surgery, First Affiliated Hospital of Xi'an Jiaotong University, Xi'an, China

**Keywords:** pyogenic liver abscess (PLA), albumin/globulin ratio, AGR, clinical characteristics, prognosis

## Abstract

Pyogenic liver abscess (PLA) remains a significant challenge for modern clinicians. Serum albumin/globulin ratio (AGR) can reflect the progress of many diseases. However, the clinical significance of AGR in PLA has not been evaluated. The aim of this study was to explore the effect of AGR on the clinical characteristic and prognosis in PLA patients. This retrospective study included 392 PLA patients who admitted to the First Affiliated Hospital of Xi'an Jiaotong University from January, 2007 to December, 2016. The medical records on admission were collected. Compared with the healthy controls and the patients with extraperitoneal infection or non-infectious liver disease, PLA patients had lower levels of AGR. The mean level of AGR in PLA patients was 1.02 ± 0.25. There were 179 (45.4%) patients with AGR > 1.02 and 213 (54.6%) patients with AGR ≤ 1.02. The baseline data and treatment plans of PLA patients with high or low AGR were comparative. However, PLA patients with a low AGR had higher body temperature, leukocytes and neutrophils, lower hemoglobin, poorer liver and coagulation function, larger abscess diameter, higher positive rate of pus culture and proportion of *Escherichia coli*, and were more susceptible to multiple bacteria. Moreover, PLA patients with a low AGR had more complications, including systemic inflammatory response syndrome (SIRS), peritoneal effusion and pleural effusion. And it also needs longer time for temperature normalization and hospital stay. In conclusion, PLA patients have lower AGR and lower AGR is associated with worse clinical manifestations, more complications and poorer prognosis. Thus, monitoring of AGR is of great clinical significance for evaluating the progress of PLA patients.

## Introduction

Pyogenic liver abscess (PLA) is a type of pyogenic infection in the liver, which can be life-threatening without appropriate treatment. PLA had a high morbidity and mortality in the past (1). The advent of modern antibiotics and intensive care techniques have dramatically reduced the mortality of PLA patients. However, the PLA incidence is still rising due to the aging of the population, the use of immunosuppressive agents, and the increase of diabetes (2). Many factors, such as malnutrition (3), diabetes (4, 5), hepatobiliary diseases (6), or accompanied by shock, low hemoglobin, high urea nitrogen, and multiple lesions in the liver (7) can influence the prognosis of PLA. Therefore, PLA remains an important challenge for today's clinicians.

The serum protein is composed by albumin (ALB) and globulin (GLO). Albumin is mainly synthesized in the liver and its level can reflect liver function. Albumin has a variety of physiological functions, such as maintaining osmotic pressure, transporting nutrient contents (fatty acids, bile acids, and cholesterol), scavenging free oxygen radicals (8–11). In addition, albumin also plays an important role in a variety of diseases (12, 13). Globulin, known as immune globulin, is mainly used to evaluate the overall status of immune and inflammatory responses (14–16).

Several recent studies have found that serum albumin/globulin ratio (AGR) can predict the prognosis of various diseases, including cancers, chronic kidney disease, heart failure, and peritoneal dialysis (17–21). As a serious infectious disease of the liver, PLA was associated with the decreased albumin and increased globulin, however, the clinical significance of AGR in PLA patients remains largely unknown. The aim of this study is to explore the effect of AGR on the clinical characteristics and prognosis of PLA patients.

## Materials and Methods

### Patients

**Figure 1:**
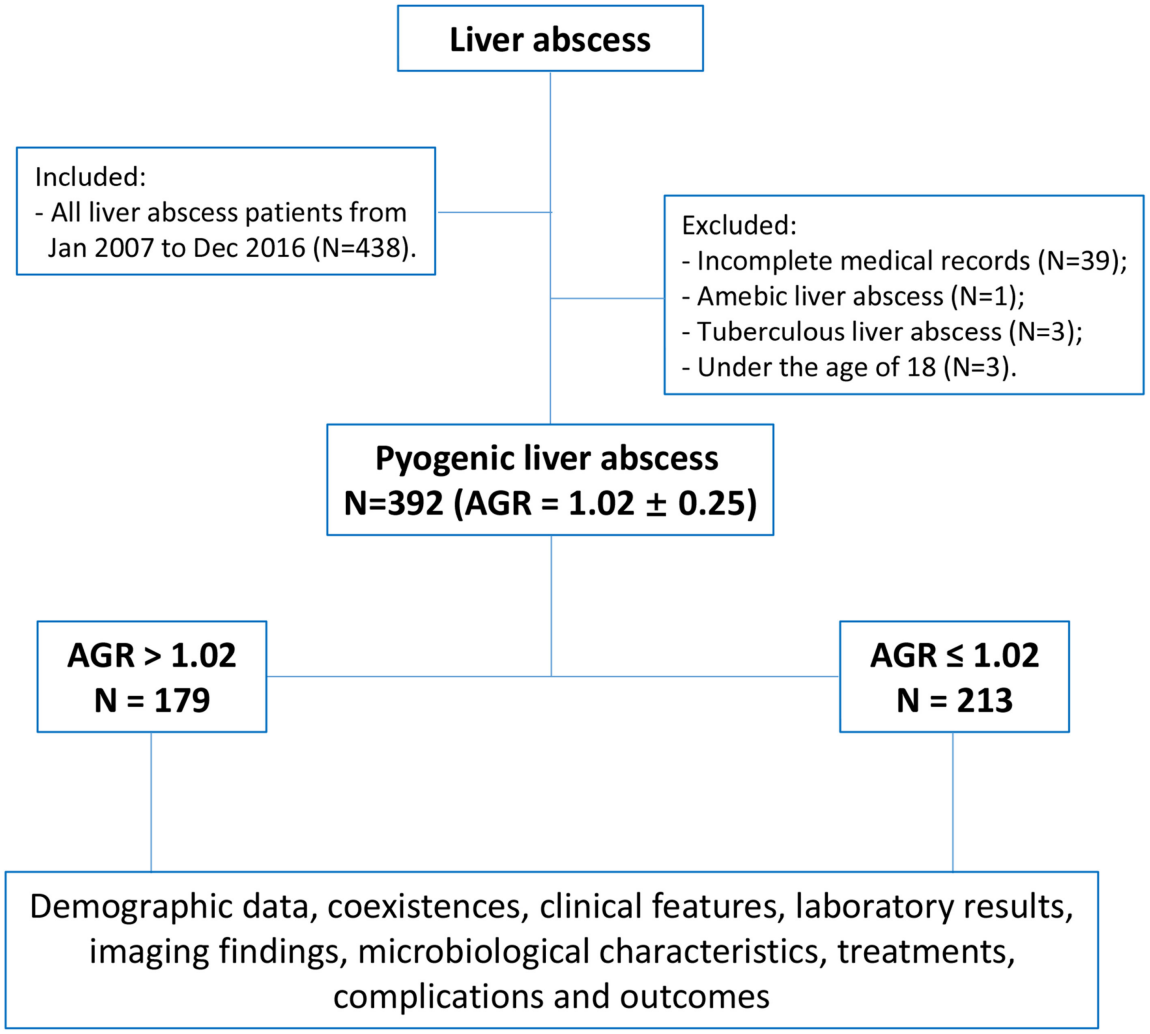
Flow chart of the study.

There were 438 patients were collected who diagnosed with liver abscess in the First Affiliated Hospital of Xi'an Jiaotong University during the 10 years from January 2007 to December 2016. The diagnosis of PLA was based on the clinical features, imaging and laboratory results, blood and pus cultures. The inclusion and exclusion criteria, and therapeutic criteria were described previously (22–24). Finally, 392 patients with pyogenic liver abscess were included in this study (Figure 1). This study was authorized by the ethic committee of the First Affiliated Hospital of Xi'an Jiaotong University (No. XJTU1AF2015LSL-057). Due to the nature of retrospective study, the patient informed consent was waived.

### Data Collection

The medical records on admission were collected, including baseline information (age, gender, height, weight, coexistences), clinical features (symptoms and signs), laboratory examinations (blood routine, liver function, kidney function, and coagulation function), imagological examination (the number, size, site of abscesses), microbiological characteristics (pus culture and blood culture), treatments (antibiotics alone, antibiotics combined with percutaneous drainage or surgical drainage), complications (systemic inflammatory response syndrome, pleural effusion, peritoneal effusion, acute respiratory distress syndrome, septic shock, spontaneous rupture of abscess, portal venous thrombosis, acute kidney injury), and outcomes (time for temperature normalization, length of hospital stay, and mortality in hospital). In addition, the AGR levels on day 1, 3, and 7 after admission and on discharge were collected.

### Statistical Analysis

Categorical variables were presented in absolute values (%) and compared by the Chi-square test or the Fisher exact test. Continuous variables were presented as mean ± standard deviation (SD) or standard error (SE) and compared by the Students *t*-test. All statistical analyses were conducted in SPSS 22.0, and the two-tailed *P* < 0.05 was considered statistically significant.

## Results

**Figure 2:**
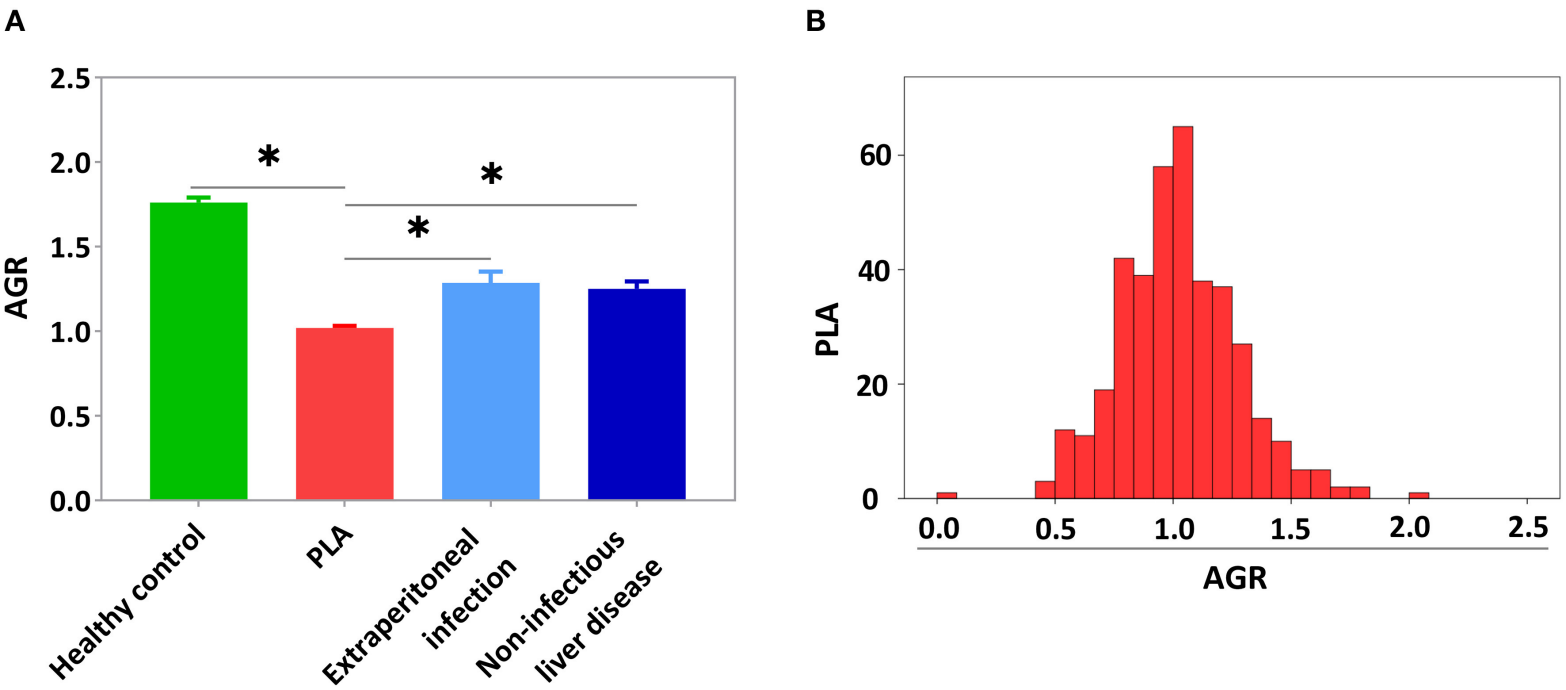
The PLA patients had lower AGR level compared with the healthy controls and the patients with extraperitoneal infection or non-infectious liver disease. **(A)** The AGR levels in healthy controls and the patients with PLA, extraperitoneal infection and non-infectious liver disease. **(B)** The AGR distribution of PLA patients. Data were presented as the mean ± SE. Differences between the groups were compared by the One-way ANOVA. *P < 0.05.

To explore the effect of PLA on serum AGR, the AGR levels of healthy controls (*n* = 50) and the patients with PLA (*n* = 392), extraperitoneal infection (*n* = 38) and non-infectious liver disease (*n* = 28) were collected and the three controls were matched with the PLA patients by age and gender (Supplementary Table 1, *P* > 0.05). However, other factors were not matched in this study. As showed in Supplementary Table 2, the most included extraperitoneal infection was pulmonary infection (26, 68.4%), and the most included non-infectious liver disease was hepatic hemangioma (16, 57.1%). Compared with healthy controls and patients with extraperitoneal infection or non-infectious liver disease, PLA patients had lower level of AGR (Figure 2A, *P* < 0.05,). There were 69.9% of PLA patients with an AGR between 0.8 and 1.3 (Figure 2B). The mean level of AGR in PLA patients was 1.02 ± 0.25. PLA patients were divided into low-AGR group (AGR ≤ 1.02) and high-AGR group (AGR > 1.02). There were 213 (54.3%) PLA patients in the low-AGR group, and 179 (45.7%) PLA patients in the high-AGR group. This cut-off value was similar to previous studies (25–27).

### Baseline Data

#### Demographic Data

As shown in the Table 1, the mean age of the patients was 57.8 ± 13.0 in the low-AGR group and 55.4 ± 13.5 in the high-AGR group. The ratio of males to females in the two groups was 108/71 and 115/98, respectively. The body mass index (BMI) of PLA patients was 22.6 ± 2.7 kg/m^2^. There was no statistically significance in age, gender, and BMI between the two groups (*P* > 0.05).

**Table 1:** Baseline data of demographic data and coexistences.

	**Total *N* = 392**	**AGR > 1.02 *N* = 179**	**AGR ≤ 1.02 *N* = 213**	***P*-value**
Age (years)	56.8 ± 13.4	55.4 ± 13.5	57.8 ± 13.0	0.068
Gender (Male/Female)	223/169	108/71	115/98	0.206
BMI (kg/m^2^)	22.6 ± 2.7	22.7 ± 2.9	22.5 ± 2.6	0.731
**Coexistences (** ***n*** **, %)**				
History of smoking	106 (27.0%)	53 (29.6%)	53 (24.9%)	0.294
History of alcohol	65 (16.6%)	36 (20.1%)	29 (13.6%)	0.085
Hypertension	77 (19.6%)	37 (20.7%)	40 (18.8%)	0.639
Diabetes mellitus	124 (31.6%)	49 (27.4%)	75 (35.2%)	0.097
Type 2 diabetes	109 (27.8%)	40 (22.3%)	69 (32.4%)	0.080
Type 1 diabetes	2 (0.5%)	1 (0.6%)	1 (0.5%)	
Undefined	13 (3.3%)	8 (4.5%)	5 (2.3%)	
Cirrhosis	16 (4.1%)	8 (4.5%)	8 (3.8%)	0.722
Viral hepatitis	29 (7.4%)	15 (8.4%)	14 (6.6%)	0.496
HBV	21 (5.4%)	14 (3.6%)	7 (3.3%)	0.055
HCV	6 (1.5%)	0 (0)	6 (2.8%)	
HBV/HCV	2 (0.5%)	1 (0.6%)	1 (0.5%)	
Hepatobiliary malignant diseases	44 (11.2%)	15 (8.4%)	29 (13.6%)	0.102
Primary hepatic carcinoma	15 (3.8%)	7 (3.9%)	8 (3.8%)	0.472
Cholangiocarcinoma	13 (3.3%)	3 (1.7%)	10 (4.7%)	
Gallbladder carcinoma	10 (2.6%)	3 (1.7%)	7 (3.3%)	
Liver metastatic carcinoma	6 (1.5%)	2 (1.1%)	4 (1.9%)	
Lithiasis	150 (38.3%)	68 (38.0%)	82 (38.5%)	0.918
Cholecystolithiasis	104 (26.5%)	51 (28.5%)	53 (24.9%)	0.476
Intrahepatic biliary stone	17 (4.3%)	5 (2.8%)	12 (5.6%)	
Extrahepatic biliary stone	12 (3.1%)	5 (2.8%)	7 (3.3%)	
Polylithiasis	17 (4.3%)	7 (3.9%)	10 (4.7%)	
Abdominal surgery history	177 (45.2%)	79 (44.1%)	98 (46.0%)	0.710
PLA history	42 (10.7%)	18 (10.1%)	24 (11.3%)	0.699

*PLA, pyogenic liver abscess; AGR, Albumin/Globulin Ratio; HBV, hepatitis B virus; HCV, hepatitis C virus; BMI, body mass index*.

#### Coexistences

There were no significant differences between the high-AGR group and the low-AGR group in terms of the coexistences, such as history of smoking and alcohol, hypertension, diabetes mellitus, cirrhosis, viral hepatitis, hepatobiliary malignant diseases, lithiasis, abdominal surgery history, and PLA history (Table 1, *P* > 0.05).

### Clinical Features, Laboratory Results, and Imaging Findings

#### Symptoms and Signs

As shown in Table 2, in terms of clinical symptoms, the two groups had similar proportions of patients with fever, chills, abdominal pain, nausea, vomiting, fatigue (*P* > 0.05). In terms of the clinical signs, respiratory rate, heart rate, and mean arterial pressure were comparable between the two groups (*P* > 0.05). However, the low-AGR group had higher temperature at admission than the high-AGR group (*P* < 0.05).

**Table 2:** Clinical features, laboratory results, and imaging findings.

	**Total *N* = 392**	**AGR > 1.02 *N* = 179**	**AGR ≤ 1.02 *N* = 213**	***P*-value**
**Symptoms and signs (*****n*****, % or mean** **±** **SD)**				
Fever	340 (87.2%)	150 (83.8%)	190 (89.2%)	0.116
Chill	197 (50.3%)	91 (50.8%)	106 (49.8%)	0.832
Abdominal pain	172 (43.9%)	75 (41.9%)	97 (45.5%)	0.469
Nausea	91 (23.2%)	38 (21.2%)	53 (24.9%)	0.393
Vomit	60 (16.3%)	25 (14.0%)	35 (16.4%)	0.499
Fatigue	69 (17.6%)	25 (14.0%)	44 (20.7%)	0.083
Temperature (°C)	37.3 ± 1.1	37.2 ± 1.1	37.4 ± 1.0	**0.048**
Respiratory rate (/min)	19.8 ± 1.8	19.9 ± 1.9	19.8 ± 1.7	0.854
Heart rate (/min)	85.1 ± 12.9	84.5 ± 13.5	85.6 ± 12.5	0.387
Mean arterial pressure (mmHg)	90.5 ± 25.3	92.2 ± 27.6	89.2 ± 23.2	0.247
**Laboratory results (mean** **±** **SD)**				
Leucocytes ( × 10^9^/L)	11.2 ± 5.7	10.2 ± 5.3	12.0 ± 6.0	**0.002**
Neutrophils ( × 10^9^/L)	9.1 ± 5.5	8.3 ± 5.1	9.8 ± 5.7	**0.007**
Lymphocytes ( × 10^9^/L)	1.3 ± 0.6	1.3 ± 0.7	1.2 ± 0.6	0.351
Hemoglobin (g/L)	111.6 ± 19.3	117.3 ± 17.7	106.9 ± 19.4	** <0.001**
Platelet count ( × 10^9^/L)	223.7 ± 125.3	221.1 ± 125.2	225.9 ± 125.7	0.705
ALT (U/L)	63.9 ± 98.7	64.5 ± 71.5	63.4 ± 117.1	0.912
AST (U/L)	54.9 ± 129.1	49.1 ± 48.8	59.8 ± 169.6	0.415
ALP (U/L)	195.0 ± 134.5	158.4 ± 103.1	225.9 ± 149.6	** <0.001**
GGT (U/L)	168.7 ± 156.5	143.8 ± 138.6	189.7 ± 167.6	**0.003**
TBIL (μmol/L)	20.9 ± 24.7	21.7 ± 30.5	20.3 ± 18.6	0.587
DBIL (μmol/L)	11.2 ± 17.4	11.3 ± 20.9	11.1 ± 13.7	0.902
Cr (umol/L)	67.1 ± 47.4	69.6 ± 50.4	65.0 ± 44.7	0.345
BUN (mmol/L)	5.2 ± 3.1	5.0 ± 2.7	5.3 ± 3.3	0.337
PT (s)	14.5 ± 1.8	14.1 ± 1.4	14.8 ± 2.1	** <0.001**
APTT (s)	39.0 ± 5.9	38.0 ± 4.9	39.7 ± 6.6	**0.004**
INR	1.16 ± 0.20	1.12 ± 0.12	1.19 ± 0.21	** <0.001**
FIB (g/L)	5.9 ± 1.9	5.8 ± 1.8	6.0 ± 1.9	0.226
**Imaging findings (*****n*****, % or mean** **±** **SD)**				
Gas formation	66 (16.8%)	23 (12.8%)	43 (20.2%)	0.053
Abscess number				
Single lesion	291 (74.2%)	136 (76.0%)	155 (72.8%)	0.469
Multiple lesions	101 (25.8%)	43 (24.0%)	58 (27.2%)	
Maximal diameter of abscess (cm)	6.7 ± 2.8	6.0 ± 2.5	7.3 ± 3.0	** <0.001**
Abscess site	*N* = 345	*N* = 154	*N* = 191	
Left lobe	46 (13.3%)	23 (14.9%)	23 (12.0%)	0.555
Right lobe	255 (73.9%)	114 (74.0%)	141 (73.8%)	
BOTH-LOBES	44 (12.8%)	17 (11.0%)	27 (14.1%)	

*PLA, pyogenic liver abscess; AGR, Albumin/Globulin Ratio; ALT, alanine transaminase; AST, aspartate transaminase; ALP, alkaline phosphatase; GGT, gamma-glutamyl transpeptidase; TBIL, total bilirubin; DBIL, direct bilirubin; Cr, creatinine; BUN, blood urea nitrogen; PT, prothrombin time; APTT, activated partial thromboplastin time; INR, international normalized ratio; FIB, fibrinogen. Bold values: P < 0.05*.

#### Laboratory Results

The blood routine, liver function, kidney function, and coagulation function on admission were collected and showed in Table 2. Compared with the high-AGR group, the low-AGR group had higher leukocytes, neutrophils, alkaline phosphatase (ALP), gamma-glutamyl transpeptidase (GGT), international standardized ratio (INR), longer prothrombin time (PT), activated partial thromboplastin time (APTT), and lower hemoglobin (*P* < 0.05), which means that the PLA patients in low-AGR group had more severe infection, poorer liver function, and coagulation function.

#### Imaging Finding

Abdominal computed tomography (CT)/ultrasound (US) was collected to determine the gas formation, number, size and site of abscesses and the results were showed in Table 2. Gas formation of abscess was comparative between the two groups (23, 12.8%/43, 20.2%, *P* > 0.05). In terms of the abscess number, single lesion was common in both groups and there was no statistical difference in the abscess number (136, 76.0%/155, 72.8%, *P* > 0.05). Abscesses in the right lobe were common and did not differ between the two groups (114, 74.0%/141, 73.8%, *P* > 0.05). However, the maximal diameter of abscess in low-AGR group was larger than that in high-AGR group (7.3 ± 3.0/6.0 ± 2.5, cm, *P* < 0.05).

### Microbiological Characteristics

#### Pus Culture

The results of pus culture from 231 PLA patients were presented in Table 3. *Klebsiella pneumoniae* was the most common pathogen in both groups (40, 33.6%/39, 34.8%, *P* > 0.05). However, the positive rate of pus culture (87, 77.7%/67, 56.3%) and the proportion of Escherichia coli (21, 18.8%/3, 2.5%) were higher in low-AGR group (*P* < 0.05), and patients in low-AGR group were more susceptible to the infection of multiple bacteria (15, 13.4%/5, 4.2%, *P* < 0.05).

**Table 3:** Microbiological characteristics.

	**Total**	**AGR > 1.02**	**AGR ≤ 1.02**	***P*-value**
**Pus culture (** ***n*** **, %)**	***N*** **=** **231**	***N*** **=** **119**	***N*** **=** **112**	
*Klebsiella pneumoniae*	79 (34.2%)	40 (33.6%)	39 (34.8%)	0.956
*Escherichia coli*	24 (10.4%)	3 (2.5%)	21 (18.8%)	** <0.001**
Enterococcus	7 (3.0%)	4 (3.4%)	3 (2.7%)	1
Streptococcus	8 (3.5%)	5 (4.2%)	3 (2.7%)	0.723
Staphylococcus	4 (1.7%)	2 (1.7%)	2 (1.8%)	1
Aerogen (Enteroaerogen)	1 (0.4%)	1 (0.8%)	0 (0)	1
Other	11 (4.8%)	7 (5.9%)	4 (3.6%)	0.410
Multiple bacteria	20 (8.7%)	5 (4.2%)	15 (13.4%)	**0.013**
No growth	77 (33.3%)	52 (43.7%)	25 (22.3%)	**0.001**
**Blood culture (** ***n*** **, %)**	***N*** **=** **163**	***N*** **=** **74**	***N*** **=** **89**	
*Klebsiella pneumoniae*	14 (8.6%)	7 (9.5%)	7 (7.9%)	0.718
*Escherichia coli*	8 (4.9%)	1 (1.4%)	7 (7.9%)	0.073
Enterococcus	2 (1.2%)	1 (1.4%)	1 (1.1%)	1
Streptococcus	4 (2.5%)	2 (2.7%)	2 (2.2%)	1
Staphylococcus	4 (2.5%)	1 (1.4%)	3 (3.4%)	0.627
Aerogen (*Clostridium perfringens*)	1 (0.6%)	1 (1.4%)	0 (0)	0.454
Other	5 (3.1%)	3 (4.1%)	2 (2.2%)	0.660
Multiple bacteria	6 (3.7%)	1 (1.4%)	5 (5.6%)	0.222
No growth	119 (73.0%)	57 (77.0%)	62 (69.7%)	0.292

*AGR, Albumin/Globulin Ratio. Bold values: P < 0.05*.

#### Blood Culture

According to the analysis of 163 PLA patients whose samples were sent for blood culture, *Klebsiella pneumoniae* was the most common pathogens in both groups (7, 9.5%/7, 7.9%, Table 3, *P* > 0.05), which was consistent with the result of pus culture. In addition, the two groups had similar positive rate and composition of positive bacteria in blood culture (Table 3, *P* > 0.05).

### Treatments, Complications, and Outcomes

#### Treatments

Patients in both groups were mainly treated with antibiotics combined with percutaneous drainage (85, 47.5%/119, 55.9%), followed by antibiotics alone (51, 28.5%/56, 26.3%), and antibiotics combined with surgical drainage was used in the least number of PLA patients (43, 24.0%/38, 17.8%). There was no statistical difference in the choice of treatments (Table 4, P > 0.05).

**Table 4:** Treatments, complications, and outcomes.

	**Total *N* = 392**	**AGR > 1.02 *N* = 179**	**AGR ≤ 1.02 *N* = 213**	***P*-value**
**Treatments (** ***n*** **, %)**				
Antibiotics alone	107 (27.3%)	51 (28.5%)	56 (26.3%)	0.194
Antibiotics combined with percutaneous drainage	204 (52.0%)	85 (47.5%)	119 (55.9%)	
Antibiotics combined with surgical drainage	81 (20.7%)	43 (24%)	38 (17.8%)	
**Complications (** ***n*** **, %)**				
SIRS	140 (35.7%)	51 (28.5%)	89 (41.8%)	**0.006**
Pleural effusion	138 (35.2%)	48 (26.8%)	90 (42.3%)	**0.001**
Peritoneal effusion	51 (13.0%)	15 (8.4%)	36 (16.9%)	**0.012**
ARDS	4 (1.0%)	2 (1.1%)	2 (0.9%)	1
Septic shock	3 (0.8%)	1 (0.6%)	2 (0.9%)	1
Spontaneous rupture of abscess	3 (0.8%)	3 (1.7%)	0 (0)	0.094
Portal venous thrombosis	3 (0.8%)	1 (0.6%)	2 (0.9%)	1
AKI	1 (0.3%)	0 (0)	1 (0.5%)	1
**Outcomes (% or mean** **±** **SD)**				
Time for temperature normalization (days)	7.4 ± 6.3	6.7 ± 6.2	8.0 ± 6.1	**0.045**
Length of hospital stay (days)	16.8 ± 9.4	15.5 ± 8.6	17.9 ± 9.9	**0.009**
Mortality in hospital	0	0	0	1

*PLA, pyogenic liver abscess; AGR, Albumin/Globulin Ratio; SIRS, systemic inflammatory response syndrome; ARDS, acute respiratory distress syndrome; AKI, acute kidney injury. Bold values: P < 0.05*.

#### Complications

The common complications are listed in Table 4, systemic inflammatory response syndrome (SIRS) (51, 28.5%/89, 41.8%), pleural effusion (48, 26.8%/90, 42.3%), peritoneal effusion (15, 8.4%/36, 16.9%) were the most common complications in two groups. However, the number of patients with SIRS, pleural effusion, peritoneal effusion was higher in low-AGR group than that in high-AGR group (*P* < 0.05). There was no statistically difference between the two groups in other less common complications, such as acute respiratory distress syndrome (ARDS), septic shock, spontaneous rupture of abscess, portal venous thrombosis, and acute kidney injury (AKI) (*P* > 0.05).

#### Outcomes

**Figure 3:**
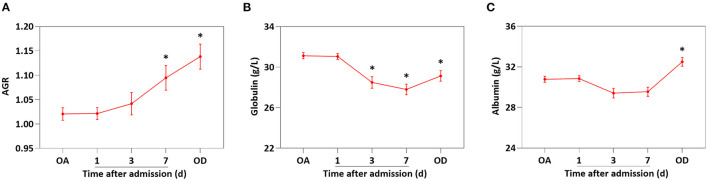
The AGR level was increased gradually after treatment in PLA patients. The levels of AGR **(A)**, globulin **(B)**, and albumin **(C)** on day 1, 3, and 7 after admission and on discharge. Data were presented as the mean ± SE. Differences between the groups were compared by the One-way ANOVA. *P < 0.05. OA, on admission; OD, on discharge.

Table 4 showed the analysis of patients' outcomes. Specifically speaking, the low-AGR group needed longer to temperature normalization and longer hospital stay than that in high-AGR group (*P* < 0.05). There were no in-hospital deaths in either group. In additon, the AGR level of PLA patients was increased gradually after treatment (Figure 3A, *P* < 0.05 vs. AGR level on admission). However, the decrease of globulin (day 3 after admission, Figure 3B) was significantly earlier than the increase of albumin (on discharge, Figure 3C).

## Discussion

In this study, the PLA patients had lower AGR level, compared with the healthy controls and the patients with extraperitoneal infection and non-infectious liver disease. The 392 PLA patients were divided into high-AGR group and low-AGR group according to the mean of AGR 1.02. There were 179 (45.4%) patients in the high-AGR group and 213 (54.6%) patients in the low-AGR group. The baseline data were consistent between the two groups. However, the low-AGR group had more serious infections, lower hemoglobin, worse liver function and coagulation function than high-AGR group. In addition, the maximal diameter of abscess in the low-AGR group was larger than that in the high-AGR group. On the other hand, the low-AGR group had higher positive rate, proportion of *Escherichia coli* and proportion of multiple bacteria. The treatments of the two groups were comparable, but the patients in low-AGR group were more likely to have complications such as SIRS, pleural effusion and peritoneal effusion. The low-AGR group also needed longer to temperature normalization and longer hospital stay than that in the high-AGR group.

PLA is one of the largest solid abscesses in the human body, it has multiple etiologies, which have been changed recently (1). Biliary diseases, including biliary stones, biliary tumors and biliary strictures, have replaced appendicitis as the most common cause of PLA (28, 29). Meanwhile, PLA with cryptogenic infection are also increasing gradually (30). Albumin/globulin ratio (AGR) is often used to guide treatment and predict prognosis in a variety of diseases. In cancers, AGR is a potential prognostic biomarker for several cancers, such as oral cavity cancer, lung cancers, nasopharyngeal cancers, renal cell carcinoma, and gastric cancers (31–35). In addition, AGR also plays a role in non-neoplastic diseases. Serum levels of ALB and GLB can be used in combination to predict the survival of patients with heart failure (18). The low AGR is associated with the significantly increased risk for subsequent vascular events in stroke patients and in subjects with clinical risk factors for stroke (17). In chronic kidney disease, AGR was suggested a simple and inexpensive measurement for detecting the risk of mortality (20). AGR has the potential to predict all-cause and cardiovascular mortality in patients on peritoneal dialysis (21). However, the effect of AGR on the clinical characteristic and prognosis in PLA patients remains unknown.

Liver is the body's albumin synthesis organ and globulin is associated with inflammation. PLA could affect the production of both albumin and globulin. In contrast, extra-abdominal infection mainly caused the increase of globulin and non-infectious liver disease mainly caused the decrease of albumin. Our results showed that AGR in both groups was lower than healthy controls, but AGR in extra-peritoneal infection and non-infectious liver disease was higher than PLA patients. This demonstrated the importance of AGR in PLA.

In the study, PLA patients was divided into two groups based on the levels of AGR. PLA patients in low-AGR group had higher body temperature, leukocytes and neutrophils on admission, which were the indication of poor prognosis (36). Moreover, the patients in low-AGR group had higher ALP, GGT, INR, longer PT, APTT, which indicating that the patients in the low-AGR group had more severe liver injury and inflammation, and worse liver function. This is not surprisingly that albumin is synthesized and secreted by hepatocytes and globulin is associated with inflammation. Patients with low AGR often have lower albumin and higher globulin, which predicts more severe liver injury and inflammation, and worse liver function. On the other hand, patients in low-AGR group had lower hemoglobin. Research has shown that hemoglobin was one of the independent prognostic factors of PLA (37). Furthermore, several studies have explored the effect of gas formation on PLA (38, 39) and gas formation of abscess was comparative between the two groups in our study. In addition, we found that the abscess diameter of the patients in the low-AGR group was significantly larger than that in the high-AGR group. Our previous study found that the larger abscess always accompanies with higher leukocytes, lower albumin, and longer time for temperature normalization (40).

With regard to microbiological characteristics, *Klebsiella pneumoniae* and *Escherichia coli* were still the most common pathogenic bacteria and *Klebsiella pneumoniae* has gradually take the place of *Escherichia coli* as the main pathogenic bacteria of PLA (41). In this study, *Klebsiella pneumoniae* was the main pathogenic bacteria, which was consistently with a previous study (42). A higher positive rate, proportion of Escherichia coli and susceptibility to multiple bacteria in pus culture were observed in the low-AGR group. In general, the factors, such as positive rate of bacterial culture and infections of Escherichia coli or multiple bacteria, always predicted a worse prognosis for PLA patients (1, 43). In addition, PLA with Aerogen infection may cause acute cholecystitis (44), however, there was no difference between the two groups.

In terms of treatments, sensitive antibiotics should be used as the preferred treatment, and combined with percutaneous drainage or surgical drainage if necessary (45). Percutaneous drainage is regarded as the preferred drainage due to its advantages of fewer complications, lower mortality, anesthetic risk, cost and shorter hospital stay (1, 46–48). Surgical drainage can be selected in the case of failure of percutaneous drainage, abscess diameter > 5 cm or multiple lesions (45, 49). In this study, antibiotics combined with percutaneous drainage was the most common treatment and the treatments were similar in both groups. Eighty-One (20.7%) PLA patients were treated with surgical drainage, which was a high percentage compared with a previous study (50). A possible reason is that the different standard (e.g., smaller abscess size) for surgical drainage was used in our study.

Many researches have shown that the low AGR is often associated with the poorer prognosis in many diseases (51–53). In the present study, although there were no in-hospital deaths in either group, patients in the low-AGR group had more complications and needed longer time for temperature normalization and hospital stay. This indicated that patients in the low-AGR group had a worse prognosis than those in the high-AGR group, which was consistent with the above results. Meanwhile, SIRS is a prevalent feature of patients with sepsis and SIRS has high sensitivity in predicting intensive care unit (ICU) admission and morbidity (54, 55). Thus, more attention needs to be paid to the low-AGR group of PLA patients. In addition, the AGR level of PLA patients was increased gradually after treatment. Therefore, AGR may be a prognostic factor for PLA patients. However, due to the retrospective nature and its inherent biases of this study, we cannot prove a cause-and-effect relationship of AGR on PLA patients, further prospective studies and larger multicenter studies are still needed to verify it. Specifically, it would be interesting to investigate if a patient, with more severe disease (lower AGR) would benefit from a more aggressive treatment in a prospective study.

In conclusion, PLA patients in low-AGR group are associated with worse clinical manifestations, more complications and poorer prognosis. AGR level may be used to measure severity and predict prognosis in PLA patients. Monitoring of AGR is of great clinical significance for the treatment and prognosis of PLA patients.

## Data Availability Statement

The raw data supporting the conclusions of this article will be made available by the authors, without undue reservation.

## Ethics Statement

This study was authorized by the ethic committee of the First Affiliated Hospital of Xi'an Jiaotong University (No. XJTU1AF2015LSL-057). Due to the nature of retrospective study, the patient informed consent was waived.

## Author Contributions

RW and JZhang designed the research. JZhang and TW wrote the manuscript. JZhang, TW, YF, MW, WL, and JZhao collected the data. JZhang, TW, and RW analyzed the data. BW, ZW, and YL interpreted the data. RW supervised the study and revised the manuscript. All authors contributed to the article and approved the submitted version.

## Funding

This work was supported by the Innovation Capacity Support Plan of Shaanxi Province (Grant No. 2020TD-040).

## Conflict of Interest

The authors declare that the research was conducted in the absence of any commercial or financial relationships that could be construed as a potential conflict of interest. The reviewer JZ declared a shared affiliation, with no collaboration, with the authors to the handling editor at the time of the review.

## Publisher's Note

All claims expressed in this article are solely those of the authors and do not necessarily represent those of their affiliated organizations, or those of the publisher, the editors and the reviewers. Any product that may be evaluated in this article, or claim that may be made by its manufacturer, is not guaranteed or endorsed by the publisher.

## Abbreviations

PLA: pyogenic liver abscess
ALB: albumin
GLO: globulin
AGR: Albumin/Globulin Ratio
CT: computed tomography
US: ultrasound
SD: standard deviation
ALT: alanine transaminase
AST: aspartate transaminase
ALP: alkaline phosphatase
GGT: gamma-glutamyl transpeptidase
TBIL: total bilirubin
DBIL: direct bilirubin
Cr: creatinine
BUN: blood urea nitrogen
PT: prothrombin time
APTT: activated partial thromboplastin time
INR: international normalized ratio
FIB: fibrinogen
SIRS: systemic inflammatory response syndrome
ARDS: acute respiratory distress syndrome
AKI: acute kidney injury
HBV: hepatitis B virus
HCV: hepatitis C virus
BMI: body mass index
ICU: intensive care unit.
