# Supplementary material for: Clinical Significance of Serum Albumin/Globulin Ratio in Patients With Pyogenic Liver Abscess
Source: Front Surg. 2021 Nov 30;8:677799. doi: 10.3389/fsurg.2021.677799 (PMC8669143; doi:10.3389/fsurg.2021.677799)
Supplement: Supplementary file 1 [file Table_1.DOCX]

**Table S1. Demographic data of each groups.**

| **Demographic data** | **PLA**  **N=392** | **Healthy individuals**  **N=50** | **Extraperitoneal infection**  **N=38** | **Non-infectious liver diseases**  **N=28** | **P value** |
| --- | --- | --- | --- | --- | --- |
| **Age (years)** | 56.8±13.4 | 55.4±3.6 | 57.0±13.2 | 57.6±9.6 | 0.889 |
| **Gender (Male/Female)** | 223/169 | 28/21 | 28/10 | 12/16 | 0.090 |

PLA, pyogenic liver abscess.

**Table S2. Clinical data of patients with extraperitoneal infection and non-infectious liver diseases.**

| **Diseases** | **Value** |
| --- | --- |
| **Extraperitoneal infection** | **N=38** |
| Pulmonary infection | 26(68.4%) |
| Limbs infection | 6(15.8%) |
| Other infection | 6(15.8%) |
| **Non-infectious liver diseases** | **N=28** |
| Hepatic hemangioma | 16(57.1%) |
| Hepatocellular carcinoma | 4(14.3%) |
| Acute hepatic injury | 4(14.3%) |
| Other liver diseases | 4(14.3%) |
